# *Letter:* Racism as a Threat to Palestinian Health Equity

**DOI:** 10.1089/heq.2024.0222

**Published:** 2025-07-16

**Authors:** Sarah McCabe

**Affiliations:** Public Health Professionals Against Antisemitism Working Group, Zion, Illinois, USA.

##  

To the Editor:

A recent article by Asi et al.^1^ claims Israelis’ anti-Palestinian racism threatens Palestinian health. The authors ignore a more obvious cause of harm to Palestinian health: Hamas’s murderous, anti-Jewish racism. The Hamas charter reads, “The Day of Judgment will not come about until Moslems fight Jews and kill them.”^2^ Because of Hamas’s terrorist attacks, Egypt and Israel restricted their borders with Gaza in 2007. Hamas leaders are billionaires, diverting aid money to enrich themselves, building tunnels that civilians cannot use as bomb shelters, and using hospitals and schools to launch rockets. Hamas’s antisemitic desire to annihilate Jews led them to murder, torture, rape, and kidnap 1,450 people on October 7, 2023. The authors sugarcoat this as “an attack by Hamas in southern Israel.” As the aggressors in the Israel-Hamas war, Hamas is responsible for the deaths of civilians they use as human shields.^3^

The authors show their own anti-Palestinian racism of low expectations by framing Palestinian leaders as helpless victims without agency. They use an America-centric/Western definition of racism, which is both inapplicable to the context and also incorrect. (They imply Israelis are “White”; the majority are people of color.) Furthermore, Palestinians are not a “race,” and Jews and Arabs are of similar genetic backgrounds; to invent anti-Palestinian “racism” is delusional.

The authors commit epistemic erasure of 3,500 years of archeological, historical, and genetic evidence showing Jews are indigenous to Israel. They ignore the Muslim Conquest, a settler-colonial empire that colonized vast areas, eradicating other indigenous ethnoreligions. They erase the approximately 900,000 Mizrahi Jews expelled from structurally racist Arab countries and Iran, which used “discriminatory beliefs” when stealing Jews’ homes and property. They erase the reason Israel has a “Law of Return”—because no country will accept Jewish refugees, as proven in the Holocaust and the expulsion of Mizrahi Jews. No Jews demand a “right to return” to Iraq, Yemen, Syria, or Lebanon. The authors demand reparations for Palestinians, forgetting the billions of dollars in aid given to them over decades or the reparations due to Mizrahi Jews.

Asi et al. state that Palestinians have better health as Israeli citizens. Rather than blaming poorer health outcomes for Palestinians in the West Bank and Gaza on the corrupt Palestinian Authority, which is responsible for their health care, instead, Asi et al., without explanation, blame “racism” by Israelis or Westerners. Authors conflate per capita health spending by the Palestinian Authority (PA) of $306 versus $3,145 in Israel, when the PA, not Israel, is responsible for health care for Palestinians in the West Bank and Gaza. Meanwhile, poorer health outcomes of Palestinian Citizens of Israel (PCI), as compared with Jewish Israelis, have been shown to be caused by preexisting poverty, higher rates of smoking, and social characteristics. Israel’s public health system has worked to improve these issues; PCI now have the longest lifespan of any Arab population in the world.^4^

Rather than framing Palestinians as helpless, we must hold them to the same expectations as any other people. We must acknowledge structural antisemitism as a health equity threat and barrier to peace.

## References

[1] Racism as a Threat to Palestinian Health Equity [Internet]. [cited 2024 Jun 22] Available from: https://www.liebertpub.com/doi/epdf/10.1089/heq.2024.0027

[2] THE COVENANT OF THE HAMAS—MAIN POINTS [Internet] [cited 2023 Dec 3]. Available from: https://irp.fas.org/world/para/docs/880818a.htm

[3] Aday S, Andžāns M, Bērziņa-Čerenkova U, et al.; NATO Strategic Communications Centre of Excellence. Hamas’ use of human shields in Gaza [Internet]. 2019. Available from: https://stratcomcoe.org/cuploads/pfiles/hamas_human_shields.pdf

[4] טאוב מ. The Health of the Arab Israeli Population[Internet]. [cited 2023 Dec 29]. Available from: https://www.taubcenter.org.il/en/research/the-health-of-the-arab-israeli-population/

